# Regional variability in phytochemical profiles and bioactivities of macerate and decoction extracts from three medicinal plants of Burkina Faso

**DOI:** 10.3389/fpls.2026.1906990

**Published:** 2026-08-04

**Authors:** Abdel Magid Arsène Ouoba, Hemayoro Sama, Harouna Soré, Abdoul-Nassiré Derra, Samson Guenné, Adama Hilou

**Affiliations:** 1 Laboratory of Biochemistry and Chemistry Applied (LABIOCA), Université Joseph KI-ZERBO, Ouagadougou, Burkina Faso; 2 Laboratory of Biochemistry, Biotechnology, Food Technology and Nutrition (LABIOTAN), Department of Biochemistry and Microbiology, Université Joseph KI-ZERBO, Ouagadougou, Burkina Faso; 3 Centre National de Recherche et de Formation sur le Paludisme (CNRFP) & Institut National de Santé Publique (INSP), Ouagadougou, Burkina Faso

**Keywords:** antibacterial activity, antioxidant activity, antiplasmodial activity, ecological variability, medicinal plants, phenolics, plant chemodiversity, secondary metabolites

## Abstract

**Introduction:**

Regional variability in secondary metabolite biosynthesis represents a critical dimension of plant chemodiversity. This study examined *Acanthospermum hispidum*, *Vitex simplicifolia*, and *Vetiveria nigritana* collected from three ecological zones of Burkina Faso (Centre-North, Centre, Cascades), focusing on phytochemical composition and bio activities.

**Methods:**

Qualitative profiling of phenolic acids and flavonoids was performed by high-performance thin-layer chromatography (HPTLC). The solvent system consisted of ethyl acetate/formic acid/glacial acetic acid/water (100/11/11/26). The total phenolic content of plant extracts was determined using the Folin–Ciocalteu method. The flavonoid content was determined using the aluminum chloride colorimetric method. The biological potential of the extracts was evaluated using three biological assays: antioxidant activities (ABTS and FRAP), antiplasmodial activity, and antibacterial activity.

**Results:**

Quantitative assays revealed pronounced differences in metabolite accumulation: *V. simplicifolia* leaves from the Centre region contained the highest polyphenol levels (450.21 ± 5.18 µg GAE/100 mg), while leaves from Cascades exhibited peak flavonoid content (269.43 ± 6.89 µg QE/100 mg). Antioxidant capacity varied accordingly, with *A. hispidum* leafy stems from Cascades showing strong ABTS activity (5.76 ± 0.32 µmol AAE/g) and *V. simplicifolia* bark from Centre-North displaying maximal FRAP reducing power (0.29 ± 0.004 µmol AAE/g). Bioactivity assays highlighted methanolic macerates of *A. hispidum* (Centre) with promising antiplasmodial activity (IC_50_ = 15.71 µg/mL), and *V. nigritana* rhizomes with potent bactericidal effects (MIC = 1.5–3 mg/mL; MBC/MIC ≤ 2). Principal Component Analysis (PCA) revealed three chemotypic clusters linking metabolite profiles to ecological origin: *V. simplicifolia* (Centre/Cascades) associated with antioxidant and antibacterial potency, *A. hispidum* (Centre) with antiplasmodial activity, and *V. nigritana* (Centre-North/Cascades) with antibacterial strength.

**Discussion:**

These findings underscore the ecological modulation of secondary metabolism and highlight the importance of regional chemodiversity for phytomedicine standardization and biodiversity conservation

## 1 Introduction

Medicinal plants remain a cornerstone of primary healthcare worldwide, particularly in sub-Saharan Africa where access to modern medicine is limited (Shey Nsagha et al., 2020). In Burkina Faso, traditional remedies are deeply embedded in cultural practices and trusted by local communities (Ouoba et al., 2023), with the World Health Organization (2002) estimating that up to 80% of populations in developing countries rely on phytotherapy. Beyond their ethnopharmacological relevance, medicinal plants provide unique models to study chemodiversity, as ecological gradients strongly modulate secondary metabolism (Riaz et al., 2023; Ceravolo et al., 2021). Plant secondary metabolism is highly responsive to ecological gradients, generating chemodiversity that underpins both biological function and therapeutic potential. Understanding how environmental variability shapes metabolite profiles is essential for linking phytochemistry to ecological adaptation and pharmacological relevance.

Burkina Faso encompasses contrasting ecological zones that directly influence plant metabolism. The Centre region (Ouagadougou area) is characterized by a sub-Sudanian climate, with annual rainfall between 700–900 mm, average temperatures of 28–30 °C, and sandy-clay soils (Zongo et al., 2023). The Cascades region (south-west) experiences a humid Sudanian climate, with higher rainfall (1 000-1 200 mm per year), mean temperatures around 27 °C, and ferruginous tropical soils rich in organic matter (Meier et al., 2025). In contrast, the Centre-Nord region lies in a transitional Sahelian zone, marked by lower rainfall (500–700 mm annually), higher average temperatures (30–35 °C), and predominantly lateritic soils (Sircoulon, 2016). These climatic and edaphic differences create distinct ecological pressures that modulate secondary metabolism, driving chemotypic differentiation within the same species (Oueslati et al., 2018; Kabore et al., 2017).

Among the medicinal plants most frequently employed in Burkina Faso, *Acanthospermum hispidum* (Ganamé et al., 2023), *Vitex simplicifolia* (Nwodo et al., 2015; Ouoba et al., 2012), and *Vetiveria nigritana* (Semde et al., 2017) are noteworthy for their traditional use against fever, gastrointestinal disorders, malaria-related symptoms, and microbial infections. These species also represent valuable systems to investigate how ecological conditions shape phenylpropanoid and terpenoid pathways, leading to distinct chemotypes. However, existing investigations remain fragmentary, often restricted to isolated compounds or single bioactivities, without integrating chemical profiling and comprehensive biological evaluation. For instance, drought and soil nutrient availability modulate phenylpropanoid pathways, altering polyphenol accumulation and antioxidant capacity. Similarly, temperature and altitude can influence terpenoid biosynthesis, leading to distinct chemotypes across regions.

Previous investigations on *Acanthospermum hispidum* have identified essential oils rich in sesquiterpenes such as β-caryophyllene, α-germacrene, bicyclogermacrene, methyl carvacrol, and α-bisabolol (Timotou et al., 2024; Alva et al., 2012), alongside polyphenolic compounds including phenolic acids and flavonoids. Its antiplasmodial, antibacterial, and antioxidant properties have been documented (Bayala et al., 2019; Yhi pênê N’do et al., 2019), yet most studies remain limited to single bioactivities or isolated metabolites. In *Vitex simplicifolia*, Bangou et al. (2019) reported diverse polyphenolic and ecdysteroid compounds, while GC-MS analyses of leaf essential oils revealed monoterpenes (myrcene, α-pinene, β-pinene), sesquiterpenes (β-caryophyllene, α-humulene, germacrene-D), and oxygenated derivatives such as linalool and humulene-1,2-epoxide (Ouoba et al., 2009). Subsequent studies demonstrated bacteriostatic, wound-healing, and antioxidant potential but without systematic comparison across ecological zones. (Bensandy et al., 2023; Ouoba et al., 2014; Ouoba et al., 2012) For *Vetiveria nigritana*, GC-MS analyses of rhizome oils highlighted prezizanoic acid, cedren-8-en-15-ol, zizanoic acid, khusian-2-ol, and khusimone as major constituents (Semde et al., 2017; Burger et al., 2017), with fractions dominated by organic acids and sesquiterpene derivatives (Champagnat et al., 2006). Antimicrobial assays confirmed broad-spectrum inhibitory activity against bacteria and fungi (Semde et al., 2017).

Taken together, these studies demonstrate the pharmacological promise of the three species but remain fragmented and geographically narrow, often restricted to single sites or specific extracts. In Burkina Faso, where ecological diversity spans the Centre, Cascades, and Centre-North regions, no integrated study has yet assessed how climatic and edaphic variability modulates phytochemical profiles and bioactivities. Addressing this gap, the present work systematically compares macerate and decoction extracts of the three species across distinct ecological zones, linking metabolite diversity to antioxidant, antibacterial, and antiplasmodial activities. This regional perspective provides a more comprehensive understanding of chemodiversity and its implications for phytomedicine standardization.

## 2 Materials and methods

### 2.1 Study area overview

**Figure 1:**
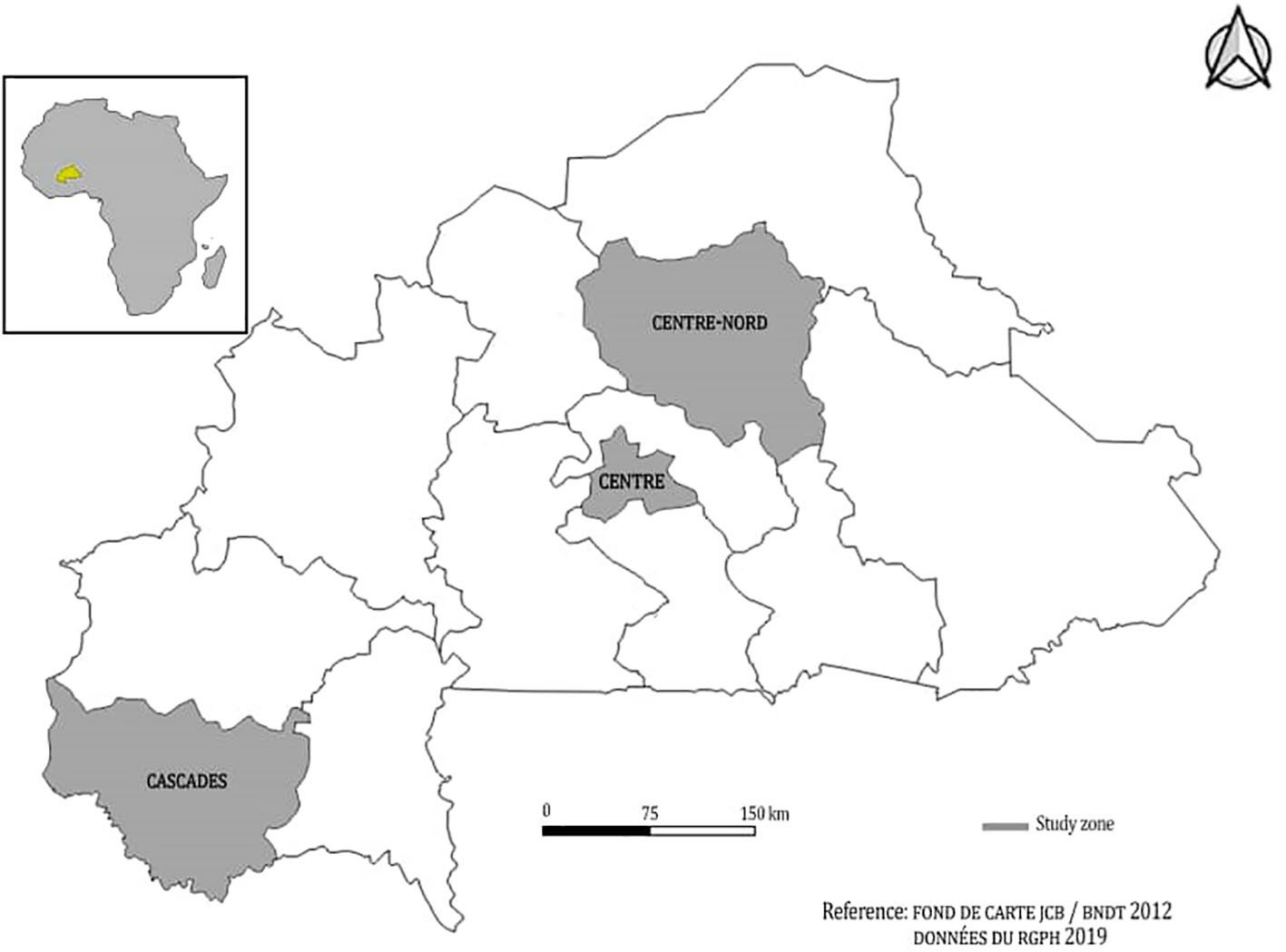
Location of the study areas.

This research was conducted in three geographically distinct regions of Burkina Faso: Centre-North, Centre, and Cascades. These regions (**Figure 1**) were selected due to the diversity of their ethnic composition and the prevalence of traditional medicine practices, thereby offering a comprehensive setting for the ethnobotanical study.

Located in the heart of West Africa, Burkina Faso covers an area of 274,200 km² and lies between 9° and 15.5° North latitude and between 6° West and 3° East longitude. It has an average elevation of 300 meters and predominantly flat terrain. Rainfall is distributed across the territory along a north-south gradient, with average annual precipitation decreasing from 1,100 mm in the south to 300 mm in the north; average daily temperatures range from 21 °C to 34 °C during the rainy season and from 17 °C to 37 °C during the dry season. The country comprises three distinct climatic zones: the Sahelian, the Sudan-Sahelian, and the Sudan-Guinean regions (Tall et al., 2019; Sivakumar and Gnoumou, 1987).

The Sahelian region (Centre-North region), situated below the 650 mm isohyet and covering areas north of the 14th parallel, is characterized by a short rainy season (July–September) marked by significant variations in rainfall distribution, severe drought driven by high evapotranspiration, and high daily and annual temperatures. Its soil cover consists of Vertisols, sub-arid brown soils, and hydromorphic soils. Its vegetation is of the steppe type (Kissou et al., 2018; Pallo and Sawadogo, 2011; Sivakumar and Gnoumou, 1987).

The Sudano-Sahelian region (Centre region), situated between the 1.000 mm and 650 mm isohyets and spanning latitudes 11°30′ to 14° North, is characterized by a rainy season lasting no more than six months (May–October). Its soil cover consists primarily of tropical ferruginous soils, weakly developed mineral soils, hydromorphic soils, and vertisols. The vegetation comprises tree and shrub savannas interspersed with steppe areas (Yaméogo, 2024; Pallo and Sawadogo, 2011; Sivakumar and Gnoumou, 1987).

The Sudanian region (Cascades region), covering areas south of 11°30’ North latitude, lies between the 1.000 mm isohyets; it is characterized by a long rainy season (May–November) and relatively low temperatures throughout the year. Its soil cover consists primarily of tropical ferruginous soils and ferrallitic soils. The vegetation is characterized mainly by tree, shrub, and woodland savannas, along with open forests (Pallo and Sawadogo, 2011; Sivakumar and Gnoumou, 1987).

### 2.2 Plant material and collection

The aerial parts of *Acanthospermum hispidum* DC., leaves of *Vitex simplicifolia* Oliv., and roots of *Vetiveria nigritana* (Benth.) Stapf. were collected from three ecologically distinct regions of Burkina Faso between August 2020 and September 2021. Voucher specimens of the collected plants were formally identified by Dr. Issouf Zerbo, Laboratory of Plant Biology and Ecology (LABEV), Joseph Ki-Zerbo University, and deposited in the publicly accessible Herbarium of Joseph Ki-Zerbo University (OUA), Burkina Faso. The following deposition numbers were assigned: *Acanthospermum hispidum* DC. (Identification number 18 000, sample number 6 965), *Vitex simplicifolia* Oliv. (Identification number 17 998, sample number 6 967), and *Vetiveria nigritana* (Benth.) Stapf. (Identification number 17 999, sample number 6 966). Plant materials were air-dried, ground into fine powder, and stored in airtight containers protected from light and humidity until use.

### 2.3 Preparation of extracts

Plant materials were shade-dried under shelter at ambient temperature (25–30 °C): leaves for 7 days and bark for 14 days. Dried samples were ground using an aluminum mortar and pestle, and the resulting powder was sieved through a stainless-steel mesh with a pore size of 0.5 mm to obtain a fine, homogeneous powder. Three types of extracts were prepared to reflect both traditional practices and standardized laboratory approaches:

Three types of extracts were prepared to reflect both traditional practices and modern approaches:

- Methanolic macerates (50 g powder in 500 ml methanol, 24 h stirring, rotary evaporation).
- Hydroalcoholic macerates (50 g powder in 80% ethanol, 24 h stirring, rotary evaporation).
- Aqueous decoctions (50 g powder boiled in 500 ml distilled water for 1 h, filtered).

Medium-pore (5 µm) filter paper was used to filter the maceration and decoction. The methanolic and hydroalcoholic extracts were concentrated by rotary evaporation under vacuum at a pressure of 200–300 mbar. The aqueous decoctions were dried in an oven at a temperature of 40 °C. The residual moisture content of the extracts was less than 5%.

All extracts have been dried at 40 °C, and stored at 4 °C until analysis. Extraction yields were calculated relative to the initial dry weight.

Methanolic and hydroalcoholic macerates were selected to maximize metabolite recovery under standardized laboratory conditions, while aqueous decoctions were included to reflect traditional practices and allow comparison between modern and ethnopharmacological approaches.

### 2.4 Phytochemical analyses

#### 2.4.1 Qualitative profiling by high-performance thin-layer chromatography

Qualitative profiling of phenolic acids and flavonoids was performed by high-performance thin-layer chromatography (HPTLC). Analyses were carried out on silica gel F254 plates (20 × 10 cm, layer thickness 0.2 mm). Extracts from all three preparation types (methanolic, hydroalcoholic, and aqueous decoctions) were applied in triplicate, with an application volume of 10 µL per lane using an automated applicator. The solvent system consisted of ethyl acetate/formic acid/glacial acetic acid/water (100/11/11/26). Plates were visualized under visible light and UV (254/365 nm) before and after spraying with NEU reagent. Reference standards (Rutin, Quercetin, and Catechin) were prepared at 1 mg/mL in methanol and applied alongside samples to aid compound identification. Scanning densitometry was performed at 280 nm and 366 nm to obtain semi-quantitative fingerprints. Medium-pore (5 µm) filter paper was used prior to application to ensure clarity of bands. This approach allowed direct comparison of chemical fingerprints across extract types and ecological origins, providing qualitative evidence of chemodiversity.

### 2.5 Quantitative assays

#### 2.5.1 Total phenolic content

The total phenolic content of plant extracts was determined using the Folin–Ciocalteu method (Singleton and Rossi, 1965). Briefly, diluted methanolic solutions of each extract were reacted with Folin–Ciocalteu reagent and sodium carbonate, and absorbance was measured at 760 nm after incubation. Gallic acid was used as the standard, and results were expressed as micrograms of gallic acid equivalents per 100 mg of extract (µg GAE/100 mg).

#### 2.5.2 Total flavonoid content

The flavonoid content of methanolic and hydroalcoholic extracts was determined using the aluminum chloride colorimetric method (Dowd, adapted by Arvouet-Grant et al., 1994). Absorbance was measured at 415 nm after complexation with AlCl_3_, and quercetin was used as the reference standard. Results were expressed as micrograms of quercetin equivalents per 100 mg of extract (µg QE/100 mg).

Although spectrophotometric assays provide reliable quantification of total phenolics and flavonoids, future studies should integrate LC–MS/MS metabolomics to identify specific compounds contributing to chemodiversity.

### 2.6 Biological activity assays

To assess therapeutic potential, extracts were subjected to three categories of bioassays:

### 2.7 Antioxidant activities

Antioxidant capacity was assessed using two complementary assays: the ABTS radical scavenging method (Re et al., 1999) and the ferric reducing antioxidant power (FRAP) assay (Hinneburg et al., 2006). Absorbances were measured at 734 nm (ABTS) and 700 nm (FRAP), respectively. Ascorbic acid served as the reference standard, and results were expressed as micromole ascorbic acid equivalents per gram of extract (µmol AAE/g).

All assays were performed in triplicate, with ascorbic acid as positive control and solvent blanks as negative controls to ensure reproducibility.

### 2.8 Antiplasmodial activity

*In vitro* antiplasmodial activity was evaluated against the chloroquine-sensitive *Plasmodium falciparum* 3D7 strain using parasitized O^+^ erythrocytes (0.5–1% parasitemia). Crude extracts were dissolved in DMSO (10 mg/mL), diluted in complete culture medium to 50 µg/mL, and incubated in 96-well plates for 72 h at 37 °C under controlled gas conditions (1% O_2_, 5% CO_2_, 94% N_2_). Parasite growth inhibition was quantified by measuring parasite lactate dehydrogenase (pLDH) activity (Makler et al., 1993). Negative (uninfected erythrocytes) and positive (infected erythrocytes without extract) controls were included. Only extracts with maximum inhibition percentages exceeding 50% are retained for the determination of the IC50. However, the inhibition percentage of the leaves of *Vitex simplicifolia* from the Centre region was not determined because the assay was inconclusive. Consequently, the determination of its IC50 was not included.

For IC_50_ determination, extracts were serially diluted in microliter plates, and parasite viability was assessed after 72 h using the pLDH assay. Chloroquine served as the reference drug. IC_50_ values were calculated by nonlinear regression (GraphPad Prism) and classified as follows: inactive (>50 µg/mL), moderately active (15–50 µg/mL), promising (5–15 µg/mL), or excellent (≤5 µg/mL). Each assay was conducted in three independent biological replicates, and IC_50_ values were statistically compared using ANOVA followed by Tukey’s *post hoc* test.

### 2.9 Antibacterial activities

#### 2.9.1 Determination of inhibition diameters and antibacterial effect

Antibacterial effects were assessed using the disc diffusion method (Arias et al., 2004), where 6 mm paper discs impregnated with plant extracts were placed on agar plates and inhibition zone diameters measured after 18–24 h incubation at 37 °C. Eight bacterial inocula were prepared with the following bacterial strain: *Escherichia coli* ATCC 25922; *Staphylococcus aureus* ATCC 25923; *Pseudomonas aeruginosa* ATCC 27653; *Escherichia coli* (clinical); *Pseudomonas fluorescens* (clinical); *Salmonella arizonae* (clinical); *Proteus mirabilis* (clinical); *Enterobacter sakazakii* (clinical). The five clinical isolates were isolated, identified, and characterized for antibiotic resistance according to the method of Tiemtoré et al. (2022) before undergoing the various tests. As for the first three strains, they are reference strains.

An inoculum of 10^6^ CFU/mL was prepared using nutrient broth and an 18- to 24-hour bacterial suspension. The strains were suspended in sterile physiological saline (0.9% NaCl), and the resulting solution was compared with a 0.5 McFarland standard.

Minimum inhibitory concentrations (MIC) and minimum bactericidal concentrations (MBC) were determined by broth microdilution (Canillac and Mourey, 2001). Serial dilutions of extracts (0.046–6 mg/mL) were prepared in microplates containing bacterial inocula (~10^5^ CFU/mL). MIC values were recorded after 18–24 h incubation at 30–37 °C, while MBC values were established by subculturing MIC wells onto Mueller–Hinton agar to identify the lowest concentration causing ≥99.99% bacterial reduction. MIC and MBC determinations were repeated in triplicate, and results were expressed as mean ± SD to account for experimental variability.

### 2.10 Statistical analysis

Data were analysed using XL-Stat software version 2016. Pearson correlation was applied to explore relationships between phytochemical content and biological activities. Principal Component Analysis (PCA) was performed on standardized variables to identify clustering patterns and compounds most associated with antibacterial and antiplasmodial activities. Significance thresholds were set at p < 0.05, and PCA robustness was validated by cross-validation to ensure reliable clustering of chemotypes.

## 3 Results

### 3.1 Phytochemical analysis

#### 3.1.1 Extraction yields

Extraction yields varied considerably depending on the plant species, the organ analyzed, and the harvest region (**Table 1**). For methanolic macerates, *Vitex simplicifolia* leaves collected in the Centre region exhibited the highest yield (17.1%), whereas the bark from the same region produced the lowest (5.9%). Hydroalcoholic macerates showed a similar variability, with the leafy stems of *Acanthospermum hispidum* from the Cascades region yielding the highest extract (22.8%), while Vitex simplicifolia leaves from the same region yielded the lowest (2.8%). In aqueous decoctions, the leafy stems of *Acanthospermum hispidum* from the Centre region provided the highest yield (19.9%), contrasting with the bark of *Vitex simplicifolia* from the Centre region, which yielded only 1.4%. This variability likely reflects differences in metabolite localization (e.g., bark vs. leaves) and ecological modulation of biosynthetic pathways, consistent with chemodiversity patterns observed in other medicinal plants.

**Table 1:** Extraction yields of macerates and decoctions.

Species	Acanthospermum hispidum			Vitex simplicifolia						Vetiveria nigritana		
Region	Centre- North	Centre	Cascades	Centre-North		Centre		Cascades		Centre- North	Centre	Cascades
Organs	Leafy stems			Barks	Leaves	Barks	Leaves	Barks	Leaves	Rhizomes		
Methanolic extract	10.6	13.2	13.9	11.4	13.7	5.9	17.1	15.9	16.1	10.1	12.4	9.1
Hydroalcoholic extract	9.7	21.4	22.8	19.9	20.4	15.7	21.0	21.4	2.8	13.2	14.9	12.3
Aqueous decoction	7.1	19.9	14.5	8.8	10.8	1.4	15.8	4.6	17.0	5.4	12.8	6.5

#### 3.1.2 Phenolics and flavonoids content

The total polyphenol and flavonoid contents also varied significantly across species, organs, and regions (**Tables 2**-**4**). In methanolic macerates, *Vitex simplicifolia* leaves from the Centre region exhibited the highest polyphenol concentration (450.21 ± 5.18 µg GAE/100 mg), while the leafy stems of *Acanthospermum hispidum* from the Centre-North region showed the lowest (137.58 ± 9.63 µg GAE/100 mg). Flavonoid levels peaked in *Vitex simplicifolia* leaves from the Cascades region (269.43 ± 6.89 µg EQ/100 mg), whereas the lowest values were recorded in *Acanthospermum hispidum* leafy stems from the Centre region (112.98 ± 5.64 µg EQ/100 mg). For hydroalcoholic macerates, *Vitex simplicifolia* leaves from the Centre region contained the highest polyphenol levels (2.92 ± 0.55 µg GAE/100 mg), while *Vetiveria nigritana* rhizomes from the Cascades region had the lowest (0.63 ± 0.09 µg GAE/100 mg). Flavonoid content was highest in *Vitex simplicifolia* leaves from the Cascades (4.87 ± 0.17 µg EQ/100 mg), contrasting with the bark from the Centre region (0.86 ± 0.04 µg EQ/100 mg). Aqueous decoctions revealed particularly high polyphenol concentrations, notably in *Vitex simplicifolia* leaves from the Cascades region (1298.06 ± 52.28 µg GAE/100 mg), while *Vetiveria nigritana* rhizomes from the Centre region showed the lowest (76.02 ± 2.46 µg GAE/100 mg). Flavonoid content was highest in *Vitex simplicifolia* bark from the Centre region (4.19 ± 0.04 µg EQ/100 mg) and lowest in *Vetiveria nigritana* rhizomes (0.50 ± 0.02 µg EQ/100 mg). Collectively, these data confirm *Vitex simplicifolia* as the most consistent source of phenolic and flavonoid compounds across extraction methods. Such inter-regional variability illustrates ecological modulation of phenylpropanoid metabolism, positioning *V. simplicifolia* as a model species for studying chemodiversity in West African flora.

**Table 2:** Polyphenol and flavonoid content of methanolic extracts.

Species	Organ used	Region	Total polyphenol µg GAE/100 mg of extract	Total flavonoid µg EQ/100 mg of extract
Acanthospermum hispidum	Leafy stem	Centre-North	137,58 ± 9,63c	200,08 ± 7,83b
		Centre	142,82 ± 3,32c	112,98 ± 5,64c
		Cascades	144,83 ± 7,56c	189,19 ± 9,89b
Vitex simplicifolia	Bark	Centre-North	380,86 ± 7,62b	124,27 ± 3,05c
	Leaf	Centre-North	370,10 ± 4,65b	188,79 ± 3,32b
	Bark	Centre	350,75 ± 8,88b	125,08 ± 6,89c
	Leaf	Centre	450,21 ± 5,18a	177,90 ± 9,89b
	Bark	Cascades	341,61 ± 5,81b	217,01 ± 6,89a
	Leaf	Cascades	264,19 ± 8,38c	269,43 ± 6,89a
Vetiveria nigritana	Rhizome	Centre-North	240,00 ± 5,58c	129,11 ± 5,64c
		Centre	236,23 ± 3,72c	125,08 ± 12,99c
		Cascades	179,24 ± 6,51c	217,01 ± 8,57a

Values followed by different letters within the same column are significantly different at p < 0.05 according to Tukey’s test.

**Table 3:** Polyphenol and flavonoid content of hydroalcoholic extract.

Species	Organ used	Region	Total polyphenol µg GAE/100 mg of extract	Total flavonoid µg EQ/100 mg of extract
Acanthospermum hispidum	Leafy stem	Centre-North	1,76 ± 0,19b	2,10 ± 0,07b
		Centre	1,95 ± 0,03b	2,08 ± 0,06b
		Cascades	2,27 ± 0,33a	1,73 ± 0,09c
Vitex simplicifolia	Bark	Centre-North	1,13 ± 0,26c	1,02 ± 0,03d
	Leaf	Centre-North	2,73 ± 1,68a	3,03 ± 0,05a
	Bark	Centre	0,81 ± 0,15c	0,86 ± 0,04d
	Leaf	Centre	2,92 ± 0,55a	3,18 ± 0,26a
	Bark	Cascades	0,86 ± 0,09c	1,54 ± 0,17c
	Leaf	Cascades	2,18 ± 0,51a	4,87 ± 0,17a
Vetiveria nigritana	Rhizome	Centre-North	1,11 ± 0,15c	1,03 ± 0,13d
		Centre	0,82 ± 0,28c	1,30 ± 0,06c
		Cascades	0,63 ± 0,09c	0,95 ± 0,12d

Values followed by different letters within the same column are significantly different at p < 0.05 according to Tukey’s test.

**Table 4:** Polyphenol and flavonoid content of aqueous decoctions.

Species	Organ used	Region	Total Phenolics µg GAE/100 mg of extract	Total flavonoid µg EQ/100 mg of extract
Acanthospermum hispidum	Leafy stem	Centre-North	473.87 ± 3.22c	2.79 ± 0,04b
		Centre	721.72 ± 3.35b	2.46 ± 0.01b
		Cascades	663.11 ± 9.17b	2.40 ± 0.01b
Vitex simplicifolia	Bark	Centre North	284.08 ± 0.93d	2.88 ± 0.01b
	Leaf	Centre-North	316.88 ± 4.05d	2.55 ± 0.00b
	Bark	Centre	393.76 ± 25.82c	4.19 ± 0.04a
	Leaf	Centre	255.59 ± 1.86d	1.34 ± 0.00c
	Bark	Cascades	737.84 ± 8.11b	2.20 ± 0.01b
	Leaf	Cascades	1298.06 ± 52.28a	2.22 ± 0.00b
Vetiveria nigritana	Rhizome	Centre-North	274.94 ± 2.46d	1.50 ± 0.17c
		Centre	76.02 ± 2.46e	0.50 ± 0.02d
		Cascades	226.55 ± 3.35d	0.94 ± 0.01d

Values followed by different letters within the same column are significantly different at p < 0.05 according to Tukey’s test.

### 3.2 TLC qualitative analysis

**Figure 2:**
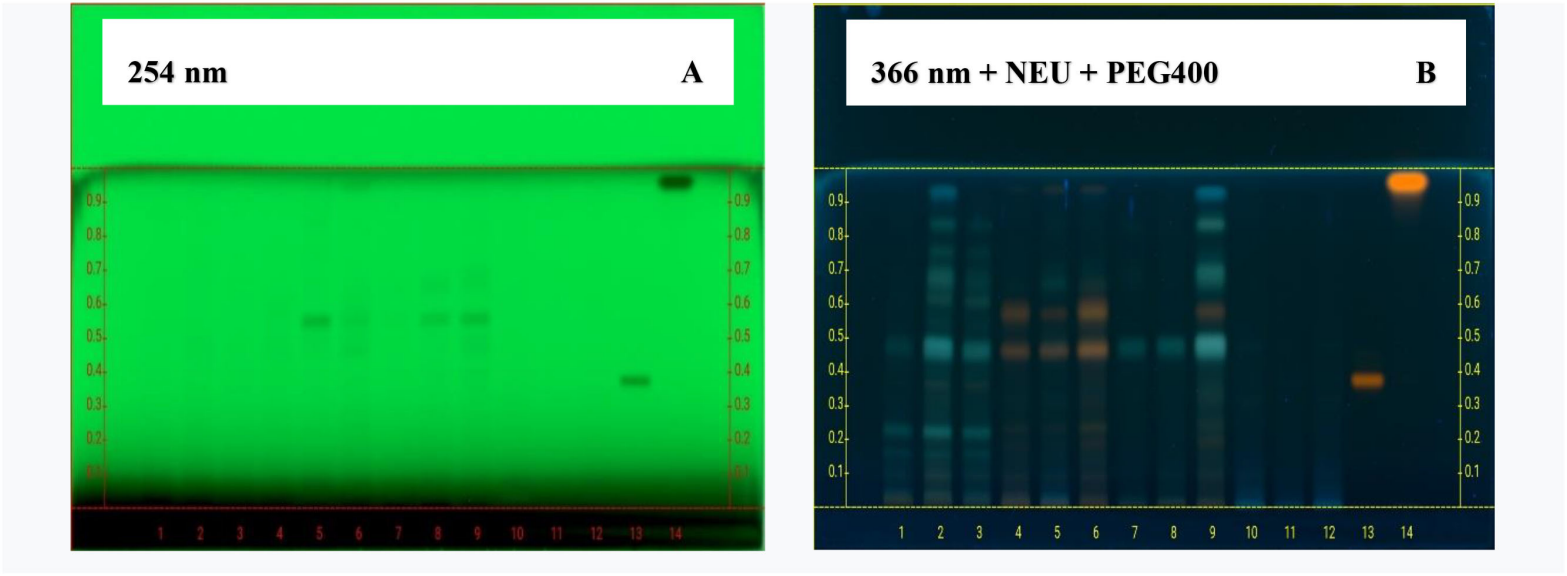
Chromatogram of flavonoids and phenolic acids in methanolic extracts. Leafy stems/1: *Acanthospermum hispidum* Centre-North; 2: *Acanthospermum hispidum* Centre; 3: *Acanthospermum hispidum* Cascades. Leaves/4: *Vitex simplicifolia* Centre-North; 5: *Vitex simplicifolia* Centre; 6: *Vitex simplicifolia* Cascades. Barks/7: *Vitex simplicifolia* Centre-North; 8: *Vitex simplicifolia* Centre; 9: *Vitex simplicifolia* Cascades. Rhizomes/10: *Vetiveria nigritana* Centre-North; 11: *Vetiveria nigritana* Centre; 12: *Vetiveria nigritana* Cascades. References/13: Rutin; 14: Quercetin; 15: Catechin; chromatogram **(A)** revealed at 254 nm; chromatogram **(B)** sprayed with NEU and PEG400 reagent and observed at 366 nm. System: Ethyl acetate/formic acid/glacial acetic acid/water (100/11/11/26).

**Figure 3:**
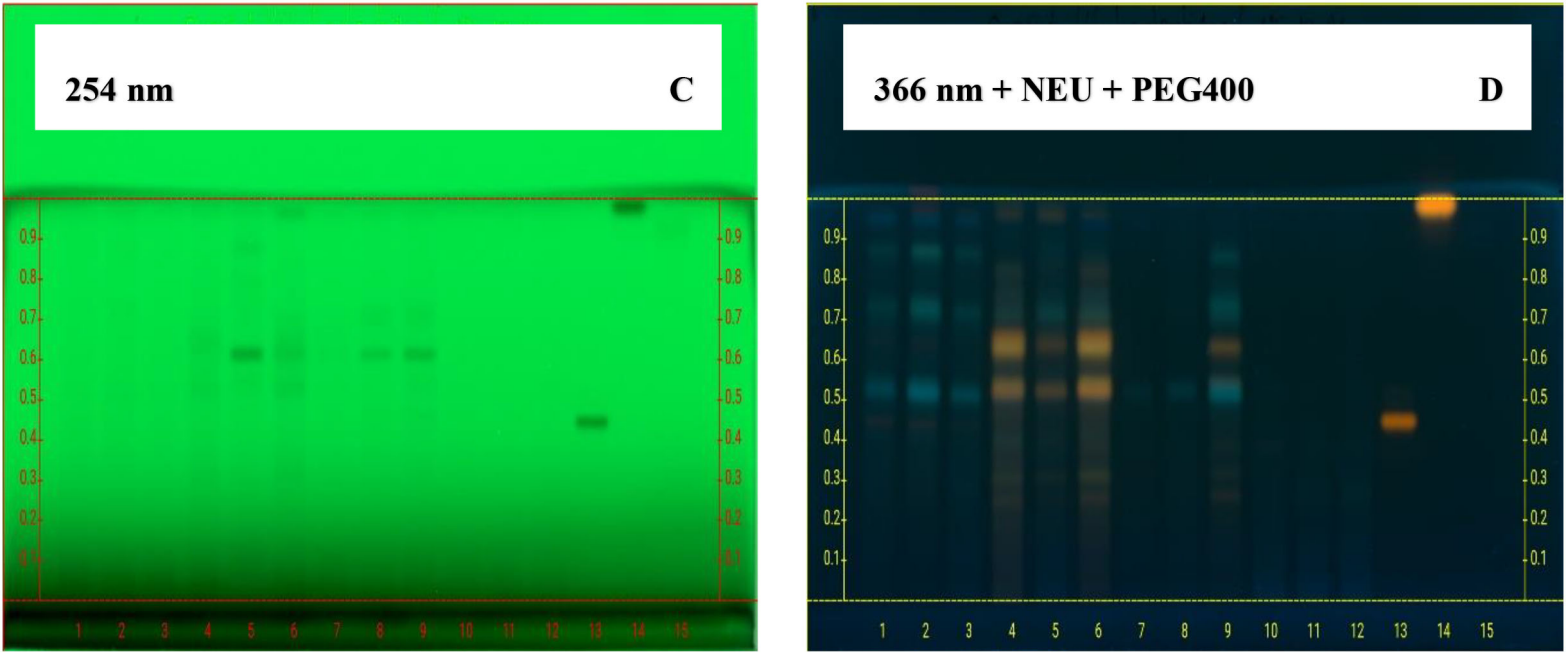
Chromatogram of flavonoids and phenol acids of hydroalcoholic extracts. Leafy stems / 1: *Acanthospermum hispidum* Centre-North; 2: *Acanthospermum hispidum* Centre; 3: *Acanthospermum hispidum* Cascades Leaves / 4: *Vitex simplicifolia* Centre-North; 5: *Vitex simplicifolia* Centre; 6: *Vitex simplicifolia* Cascades Barks / 7: *Vitex simplicifolia* Centre-North; 8: *Vitex simplicifolia* Centre; 9: *Vitex simplicifolia* Cascades Rhizomes / 10: *Vetiveria nigritana* Centre-North; 11: *Vetiveria nigritana* Centre; 12: *Vetiveria nigritana* Cascades References / 13: Rutin; 14: Quercetin; 15: Catechin; chromatogram **(C)** revealed at 254 nm; chromatogram **(D)** sprayed with NEU and PEG400 reagent and observed at 366 nm. System: Ethyl acetate/formic acid/glacial acetic acid/water (100/11/11/26).

**Figure 4:**
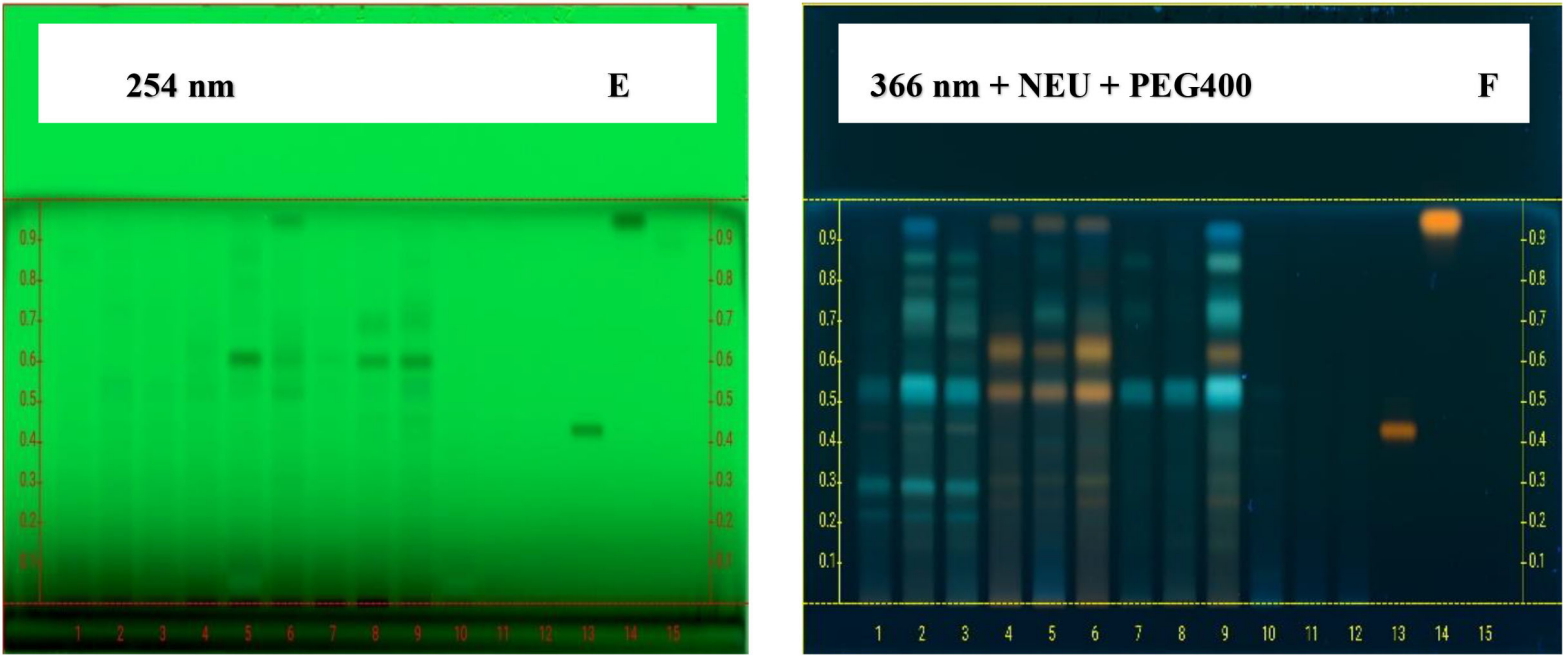
Chromatogram of flavonoids and phenol acids of aqueous decoctions. Leafy stems / 1: *Acanthospermum hispidum* Centre-North; 2: *Acanthospermum hispidum* Centre; 3: *Acanthospermum hispidum* Cascades Leaves / 4: *Vitex simplicifolia* Centre-North; 5: *Vitex simplicifolia* Centre; 6: *Vitex simplicifolia* Cascades Barks / 7: *Vitex simplicifolia* Centre-North; 8: *Vitex simplicifolia* Centre; 9: *Vitex simplicifolia* Cascades Rhizomes / 10: *Vetiveria nigritana* Centre-North; 11: *Vetiveria nigritana* Centre; 12: *Vetiveria nigritana* Cascades References / 13: Rutin; 14: Quercetin; 15: Catechin; chromatogram **(E)** revealed at 254 nm; chromatogram **(F)** sprayed with NEU and PEG400 reagent and observed at 366 nm. System: Ethyl acetate/formic acid/glacial acetic acid/water (100/11/11/26).

High-Performance Thin Layer Chromatography (HPTLC) revealed distinct phenolic acid and flavonoid profiles across extracts (**Figures 2**–**4**). Methanolic macerates of *Acanthospermum hispidum* and *Vitex simplicifolia* displayed clear regional differences, with samples from the Centre and Cascades regions showing a greater diversity of spots. Hydroalcoholic macerates were generally homogeneous, except for *Vitex simplicifolia* bark extracts, which exhibited richer profiles in the Cascades region. Decoctions highlighted stable profiles for *Vetiveria nigritana* rhizomes, while *Vitex simplicifolia* bark from Cascades and leaves from the Centre region showed marked variability. These qualitative differences reinforce the role of geographical origin in shaping phytochemical diversity.

### 3.3 Antioxidant activities

The antioxidant potential of macerates and decoctions, assessed by ABTS radical scavenging and FRAP reducing assays, varied significantly with species, plant organ, and harvest region (**Tables 12**–**13**). For methanolic macerates, the leafy stems of *Acanthospermum hispidum* from the Cascades region exhibited the strongest ABTS activity (5.76 ± 0.32 µmol EAA/g), while the bark of *Vitex simplicifolia* from the same region showed the lowest (4.44 ± 0.05 µmol EAA/g). In contrast, FRAP activity was highest in *Vitex simplicifolia* bark from the Centre-North (0.29 ± 0.004 µmol EAA/g) and lowest in *Vetiveria nigritana* rhizomes from the Centre-North (0.07 ± 0.01 µmol EAA/g).

**Table 5:** *In vitro* antioxidant activities of methanolic macerates.

Plant species	Organ used	Region	ABTS (µmol EAA/g of extract)	FRAP (µmol EAA/g of extract)
Acanthospermum hispidum	Leafy stem	Centre-North	4.52 ± 0.46c	0.12 ± 0.004c
		Centre	4.74 ± 0.28c	0.13 ± 0.006c
		Cascades	5.76 ± 0.32a	0.18 ± 0.003b
Vitex simplicifolia	Bark	Centre-North	4.75 ± 0.11c	0.29 ± 0.004a
	Leaf	Centre-North	5.11 ± 0.13b	0.18 ± 0.002b
	Bark	Centre	4.84 ± 0.29c	0.14 ± 0.006c
	Leaf	Centre	4.69 ± 0.03c	0.19 ± 0.003b
	Bark	Cascades	4.44 ± 0.05d	0.14 ± 0.006c
	Leaf	Cascades	4.59 ± 0.26d	0.19 ± 0.005b
Vetiveria nigritana	Rhizomes	Centre-North	4.69 ± 0.58c	0.07 ± 0.01d
		Centre	4.61 ± 0.07c	0.11 ± 0.007c
		Cascades	4.83 ± 0.78c	0.08 ± 0.003d

Values followed by different letters within the same column are significantly different at p < 0.05 according to Tukey’s test.

**Table 6:** *In vitro* antioxidant activities of hydroalcoholic macerates.

Plant species	Organ used	Region	ABTS (µmol EAA/g of extract)	FRAP (µmol EAA/g of extract)
Acanthospermum hispidum	Leafy stem	Centre-North	4.22 ± 0.23c	0.05 ± 0.003c
		Centre	4.49 ± 0.09b	0.06 ± 0.001c
		Cascades	4.60 ± 0.11b	0.05 ± 0.003c
Vitex simplicifolia	Bark	Centre-North	4.44 ± 0.20b	0.08 ± 0.004b
	Leaf	Centre-North	4.65 ± 0.02b	0.10 ± 0.005b
	Bark	Centre	4.70 ± 0.26b	0.06 ± 0.004c
	Leaf	Centre	4.38 ± 0.06c	0.21 ± 0.006a
	Bark	Cascades	4.55 ± 0.02b	0.06 ± 0.005c
	Leaf	Cascades	4.88 ± 0.79a	0.10 ± 0.003b
Vetiveria nigritana	Rhizomes	Centre-North	3.79 ± 0.37d	0.04 ± 0.003c
		Centre	5.47 ± 0.83a	0.05 ± 0.004c
		Cascades	4.17 ± 0.41c	0.04 ± 0.003c

Values followed by different letters within the same column are significantly different at p < 0.05 according to Tukey’s test.

**Table 7:** *In vitro* antioxidant activities of aqueous decoctions.

Plant species	Organ used	Region	ABTS (µmol EAA/g of extract)	FRAP (µmol EAA/g of extract)
Acanthospermum hispidum	Leafy stem	Centre-North	4.93 ± 0.03a	0.83 ± 0.002b
		Centre	4.88 ± 0.01a	0.31 ± 0.002c
		Cascades	4.88 ± 0.01a	0.97 ± 0.001a
Vitex simplicifolia	Bark	Centre-North	4.91 ± 0.01a	0.74 ± 0.005b
	Leaf	Centre-North	4.90 ± 0.03a	0.30 ± 0.000c
	Bark	Centre	4.94 ± 0.13a	0.60 ± 0.000b
	Leaf	Centre	4.85 ± 0.05a	0.38 ± 0.000c
	Bark	Cascades	4.85 ± 0.00a	0.45 ± 0.020c
	Leaf	Cascades	4.92 ± 0.02a	0.46 ± 0.005c
Vetiveria nigritana	Rhizomes	Centre-North	4.70 ± 0.02b	0.21 ± 0.016d
		Centre	4.88 ± 0.03a	0.03 ± 0.001e
		Cascades	4.84 ± 0.01a	0.24 ± 0.011d

Values followed by different letters within the same column are significantly different at p < 0.05 according to Tukey’s test.

Hydroalcoholic macerates revealed strong ABTS activity in Vetiveria nigritana rhizomes from the Centre (5.47 ± 0.83 µmol EAA/g), whereas the same organ from the Centre-North displayed the weakest (3.79 ± 0.37 µmol EAA/g). FRAP activity peaked in Vitex simplicifolia leaves from the Centre (0.21 ± 0.006 µmol EAA/g).

Aqueous decoctions showed consistently high ABTS values across species, with Vitex simplicifolia bark from the Centre reaching 4.94 ± 0.13 µmol EAA/g. FRAP activity was most pronounced in Acanthospermum hispidum leafy stems from the Cascades (0.97 ± 0.001 µmol EAA/g), while Vetiveria nigritana rhizomes from the Centre yielded the lowest (0.03 ± 0.001 µmol EAA/g). The strong association between polyphenol/flavonoid levels and antioxidant indices confirms that ecological gradients not only alter metabolite abundance but also functional antioxidant capacity, reinforcing the chemodiversity framework.

### 3.4 Antiplasmodial activities

*In vitro* screening against *Plasmodium falciparum* (3D7 strain) revealed that only methanolic macerates achieved inhibition rates above 50% at 50 µg/mL (**Table 8**). **Tables 9** and **10** illustrate the inhibition percentages of hydroalcoholic macerates and aqueous decoctions, respectively. The most active extracts were *Vitex simplicifolia* leaves from the Cascades (72.47%), *Acanthospermum hispidum* leafy stems from the Centre (62.09%), *Vitex simplicifolia* bark from the Centre (61.77%), and *Vitex simplicifolia* leaves from the Centre-North (61.29%). Hydroalcoholic macerates and aqueous decoctions generally showed weaker inhibition (<50%).

**Table 8:** *In vitro* screening of the antiplasmodial activity of methanolic macerates.

Strain tested 3D7				
Plant species	Organ used	Region	Concentration (µg/ml)	Inhibition percentage (%)
Acanthospermum hispidum	Leafy stem	Centre-North	50	**57.143**
		Centre	50	**62.091**
		Cascades	50	**59.856**
Vitex simplicifolia	Bark	Centre-North	50	46.064
	Leaf	Centre-North	50	**61.293**
	Bark	Centre	50	**61.772**
	Leaf	Centre	50	Not determined
	Bark	Cascades	50	**56.185**
	Leaf	Cascades	50	**72.466**
Vetiveria nigritana	Rhizome	Centre-North	50	46.569
		Centre	50	**53.551**
		Cascades	50	**55.706**

Values in **bold** indicate inhibition percentages greater than 50 %, considered biologically relevant thresholds for antiplasmodial activity.

**Table 9:** *In vitro* screening of the antiplasmodial activity of hydroalcoholic macerates.

Strain tested 3D7				
Plant species	Organ used	Region	Concentration (µg/ml)	Inhibition percentage (%)
Acanthospermum hispidum	Leafy stem	Centre-North	50	18.308
		Centre	50	25.884
		Cascades	50	25.126
Vitex simplicifolia	Bark	Centre-North	50	10.985
	Leaf	Centre-North	50	42.803
	Bark	Centre	50	13.005
	Leafy	Centre	50	23.864
	Bark	Cascades	50	Not determined
	Leafy	Cascades	50	17.677
Vetiveria nigritana	Rhizome	Centre-North	50	00
		Centre	50	15.783
		Cascades	50	1.515

No value is greater than or equal to 50%, the value considered to be the biologically relevant threshold for antiplasmodial activity.

**Table 10:** *In vitro* screening of the antiplasmodial activity of decoctions macerates.

Strain tested 3D7				
Plant species	Organ used	Region	Concentration (µg/ml)	Inhibition percentage (%)
Acanthospermum hispidum	leafy stem	Centre-North	50	15.025
		Centre	50	27.652
		Cascades	50	44.192
Vitex simplicifolia	Bark	Centre-North	50	Not determined
	Leaf	Centre-North	50	43.056
	Bark	Centre	50	15.783
	Leaf	Centre	50	28.283
	Bark	Cascades	50	21.97
	Leaf	Cascades	50	33.586
Vetiveria nigritana	Rhizome	Centre-North	50	22.475
		Centre	50	28.788
		Cascades	50	Not determined

No value is greater than or equal to 50%, the value considered to be the biologically relevant threshold for antiplasmodial activity.

IC50 determinations confirmed moderate antiplasmodial activity for selected methanolic extracts (**Table 11**). The lowest IC50 was observed in *Acanthospermum hispidum* leafy stems from the Centre (15.71 µg/mL), followed by *Vitex simplicifolia* leaves from the Cascades (25.04 µg/mL), *Vitex simplicifolia* leaves from the Centre-North (42.06 µg/mL), and bark from the Centre (47.98 µg/mL). Although these values are higher than the reference drug chloroquine (IC50 = 0.78 µg/mL), they indicate promising activity warranting further investigation.

**Table 11:** Inhibitory concentration (IC_50_) of the antiplasmodial activity of methanolic macerates.

Plant species	Organ used	Region	50% inhibitory concentration (IC50)
Acanthospermum hispidum	leafy stem	Centre	15.71
Vitex simplicifolia	Bark	Centre	47.98
	Leaf	Centre-North	42.06
	Leaf	Cascades	25.04
Chloroquine			0.78

IC50 determinations confirmed moderate antiplasmodial activity for selected methanolic extracts (**Table 11**). The lowest IC50 was observed in *Acanthospermum hispidum* leafy stems from the Centre (15.71 µg/mL), followed by *Vitex simplicifolia* leaves from the Cascades (25.04 µg/mL), *Vitex simplicifolia* leaves from the Centre-North (42.06 µg/mL), and bark from the Centre (47.98 µg/mL). Although these values are higher than the reference drug chloroquine (IC50 = 0.78 µg/mL), they indicate promising activity warranting further investigation.

### 3.5 Antibacterial activities

The antibacterial assays demonstrated that inhibition zones, MICs, and MBCs varied across species, organs, and regions (**Table 12**). Methanolic extracts produced inhibition zones ranging from 9 to 20 mm, hydroalcoholic extracts from 9 to 19 mm, and aqueous decoctions from 9 to 12 mm. Reference antibiotics (Ceftazidime and Ciprofloxacin) showed broader ranges (9–38 mm), as expected.

**Table 12:** Summary of inhibition zones.

Species	Extract type	Representative strains	Range of inhibition diameter (mm)	Reference antibiotics (mm)	Most active organs/regions
*A. hispidum*	Methanolic	*E. coli, S. aureus, P. aeruginosa, S. arizonae, P. mirabilis, E. sakazakii*	9–18	CAZ 10–26; CIP 17–38	Leafy stems (Centre, Cascades)
	Hydroalcoholic	*E. coli, S. arizonae, E. sakazakii*	9–19	CAZ 9–31; CIP 28–32	Leafy stems (Centre-North, Cascades)
	Decoction	*E. coli, P. mirabils, E. sakazakii*	9–10	CAZ 9–28; CIP 27–33	Leafy stems (Cascades)
*V. simplicifolia*	Methanolic	*E. coli, S. aureus, P. aeruginosa, S. arizonae, P. mirabilis, E. sakazakii*	9–20	CAZ 10–26; CIP 17–38	Leaves (Centre-North, Cascades)
	Hydroalcoholic	*E. coli, P. aeruginosa, E. sakazakii*	9–15	CAZ 9–31; CIP 28–32	Bark (Centre)
	Decoction	*E. coli, P. fluorescens, S. arizonae, P. mirabilis, E. sakazakii*	9–12	CAZ 9–28; CIP 27–33	Leaves (Centre, Cascades)
*V. nigritana*	Methanolic	*E. coli, S. aureus, P. aeruginosa, P. fluorencens, S. arizonae, P. mirabilis, E. sakazakii*	9–21	CAZ 10–26; CIP 17–38	Rhizomes (Centre, Centre-North)
	Hydroalcoholic	*E. coli, S. aureus, S. arizonae, E. sakazakii*	9–14	CAZ 9–23; CIP 28–32	Rhizomes (Centre-North)
	Decoction	*E. coli, S. arizonae, P. mirabilis, E. sakazakii*	9–11	CAZ 9–28; CIP 27–33	Rhizomes (Centre-North)

All raw values, present the range of inhibition diameters per extract type and species.

MIC and MBC analyses revealed strong antibacterial activity in Vetiveria nigritana rhizomes across all regions, with MICs as low as 1.5 mg/mL and MBC/MIC ratios ≤2, indicating bactericidal effects. Extracts of Vitex simplicifolia bark and leaves, as well as Acanthospermum hispidum leafy stems, also displayed moderate activity (MICs 1.5–6 mg/mL). Hydroalcoholic and aqueous extracts showed similar patterns, with most MBC/MIC ratios ≤2, confirming bactericidal properties.

**Table 13:** MIC, MBC, MBC/MIC of extracts.

Species	Extract type	Region (s)	Representative strains	MIC range (mg/mL)	MBC range (mg/mL)	MBC/MIC	Effect
*A. hispidum*	Methanolic	Centre, Centre-North, Cascades	*E. coli, S. aureus, P. aeruginosa, S. arizonae, P. mirabilis, E. sakazakii*	1.5–6	1.5–6	≤ 2	Bactericidal
	Hydroalcoholic	Centre, Centre-North, Cascades	*E. coli, S. arizonae, E. sakazakii*	1.5–6	1.5–6	≤ 2	Bactericidal
	Decoction	Centre, Centre-North, Cascades	*E. coli, P. mirabilis, E. sakazakii*	3–6	1.5–3	≤ 2	Bactericidal
*V. simplicifolia*	Methanolic	Centre, Centre-North, Cascades	*E. coli, S. aureus, P. aeruginosa, S. arizonae, P. mirabilis, E. sakazakii*	1.5–6	1.5–6	≤ 2	Bactericidal
	Hydroalcoholic	Centre, Centre-North, Cascades	*E. coli, P. aeruginosa, E. sakazakii*	1.5–6	1.5–6	≤ 2	Bactericidal
	Decoction	Centre, Centre-North, Cascades	*E. coli, P. fluorencens, S. arizonae, P. mirabilis, E. sakazakii*	1.5–6	1.5–6	≤ 2	Bactericidal
*V. nigritana*	Methanolic	Centre, Centre-North, Cascades	*E. coli, S. aureus, P. aeruginosa, P. fluorencens, S. arizonae, P. mirabilis, E. sakazakii*	1.5–3	1.5	≤ 2	Bactericidal
	Hydroalcoholic	Centre, Centre-North, Cascades	*E. coli, S. aureus, S. arizonae, E. sakazakii*	1.5–6	1.5–6	≤ 2	Bactericidal
	Decoction	Centre, Centre-North, Cascades	*E. coli, S. arizonae, P. mirabilis, E.sakazakii*	3–6	1.5–6	≤ 2	Bactericidal

### 3.5 Correlation matrix analysis

The correlation matrix allowed us to assess the relationships between phytochemical composition and biological activities according to the species studied and the harvest regions.

For methanolic macerates, a negative and almost zero correlation (r = -0.056) was observed between total polyphenol and flavonoid content; a weak negative correlation (r = -0.373) was found between total polyphenol content and the ABTS antioxidant index. However, there was a moderate positive correlation (r = 0.532) between total polyphenol content and the FRAP antioxidant index, and an almost zero correlation between total flavonoid content and the ABTS and FRAP antioxidant indices (r = 0.049; 0.061). The correlation between the ABTS and FRAP antioxidant indices was almost zero. Polyphenols appear to contribute to both antioxidant and reducing capacity (**Table 14**).

**Table 14:** Correlation matrix between chemical compositions and antioxidant activities of methanolic macerates.

Variables	Total phenolics	Total flavonoids	ABTS	FRAP
Total Phenolics	**1**			
Total flavonoids	-0.056	**1**		
ABTS	-0.373	0.049	**1**	
FRAP	0.532	0.061	0.133	**1**

The value 1 shows that each variable is perfectly correlated with itself.

In the case of hydroalcoholic macerates, a strong positive correlation (r = 0.753) is observed between the total polyphenol and flavonoid content, and between these polyphenolic compounds and the FRAP antioxidant indices (r = 0.708). In contrast, the correlations between total flavonoid content and the ABTS and FRAP antioxidant indices are relatively weak to moderate (r = 0.225; 0.607). The correlation between the ABTS and FRAP antioxidant indices is moderate (r = 0.051). Thus, polyphenols appear to contribute significantly to FRAP reducing capacity; however, their impact on free radical scavenging capacity is very weak or even negligible (**Table 15**).

**Table 15:** Correlation matrix between chemical compositions and antioxidant activities of hydroalcoholic macerates.

Variables	Total phenolics	Total flavonoids	ABTS	FRAP
Total phenolics	**1**			
Total flavonoids	0.753	**1**		
ABTS	-0.001	0.225	**1**	
FRAP	0.708	0.607	0.051	**1**

The value 1 shows that each variable is perfectly correlated with itself.

Finally, for aqueous decoctions, a weak correlation (r = 0.302) was observed between the total polyphenol and flavonoid content, and between total polyphenols and the ABTS (r = 0.275) and FRAP (r = 0.305) antioxidant indices. However, there was a relatively moderate correlation between total flavonoid content and the ABTS (r = 0.547) and FRAP (r = 0.647) antioxidant indices. The correlation between the ABTS and FRAP antioxidant indices was also moderate. Polyphenols did not appear to influence free radical scavenging and reducing activity. However, total flavonoids did contribute to free radical scavenging and reducing capacities (**Table 16**).

**Table 16:** Correlation matrix between chemical compositions and antioxidant activities of aqueous decoctions.

Variables	Total phenolics	Total flavonoids	ABTS	FRAP
Total phenolics	**1**			
Total Flavonoids	0.302	**1**		
ABTS	0.275	0.547	**1**	
FRAP	0.305	0.647	0.458	**1**

The value 1 shows that each variable is perfectly correlated with itself.

### 3.6 Principal component analysis

The PCA was performed to integrate phytochemical parameters with antioxidant activity data across extraction methods.

**Figure 5:**
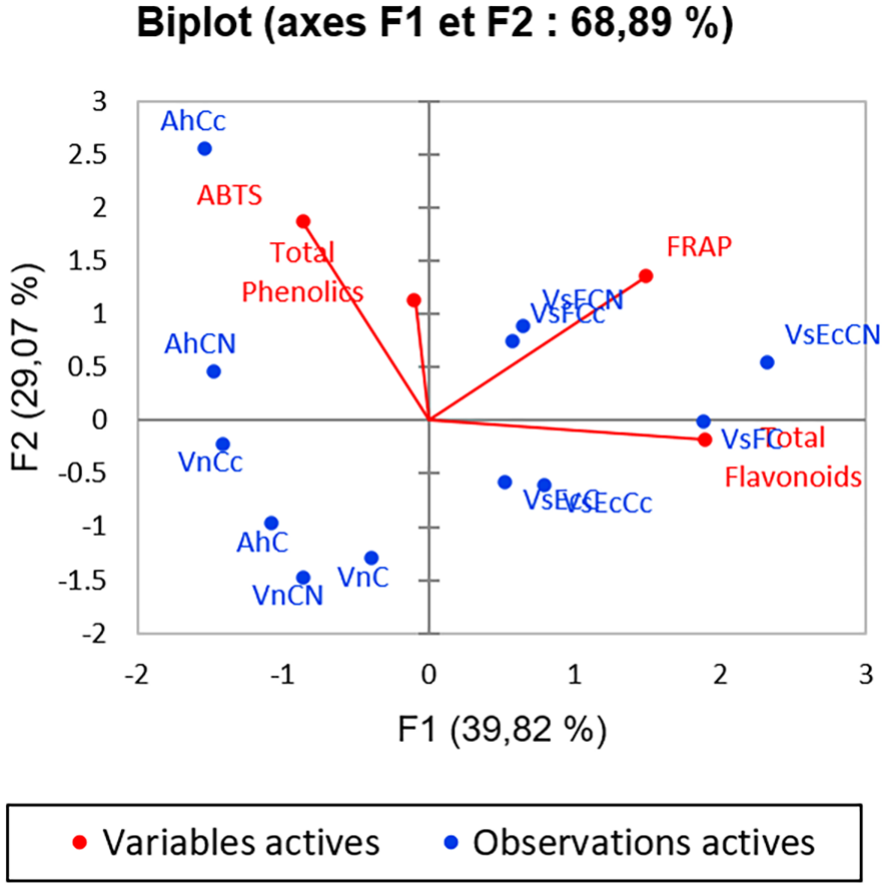
Principal component analysis (PCA) of the phytochemical profiles of methanolic macerates and their antioxidant activities.

**Figure 6:**
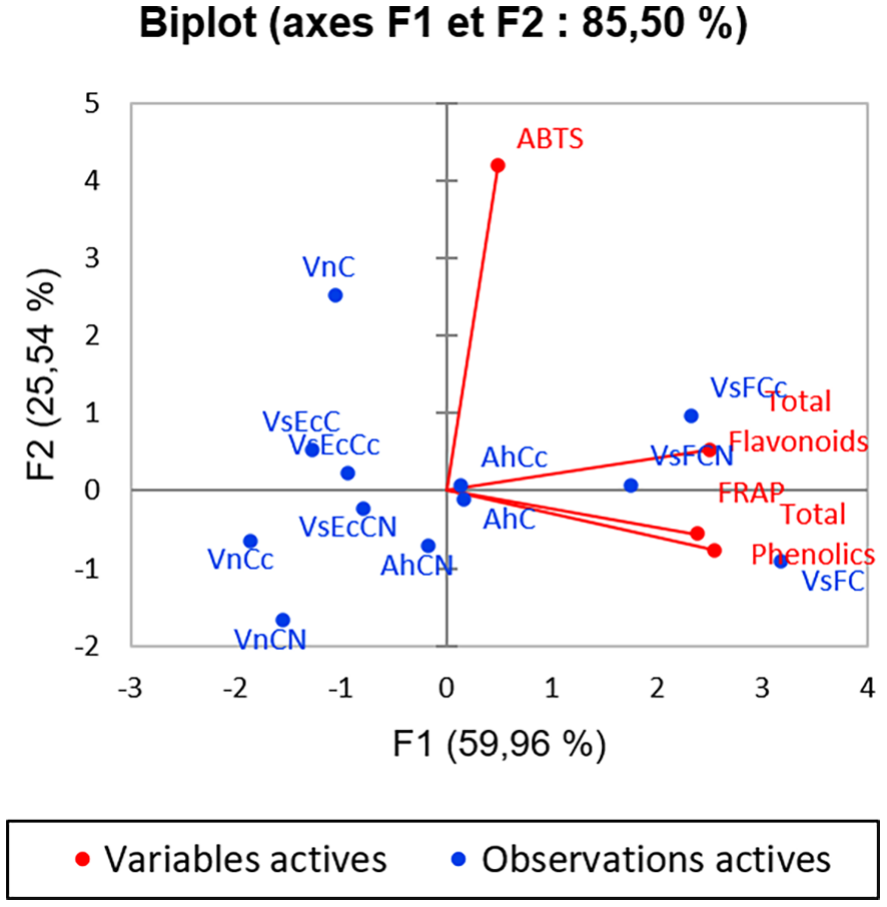
Principal component analysis (PCA) of the phytochemical profiles of hydroalcoholic macerates and their antioxidant activities.

**Figure 7:**
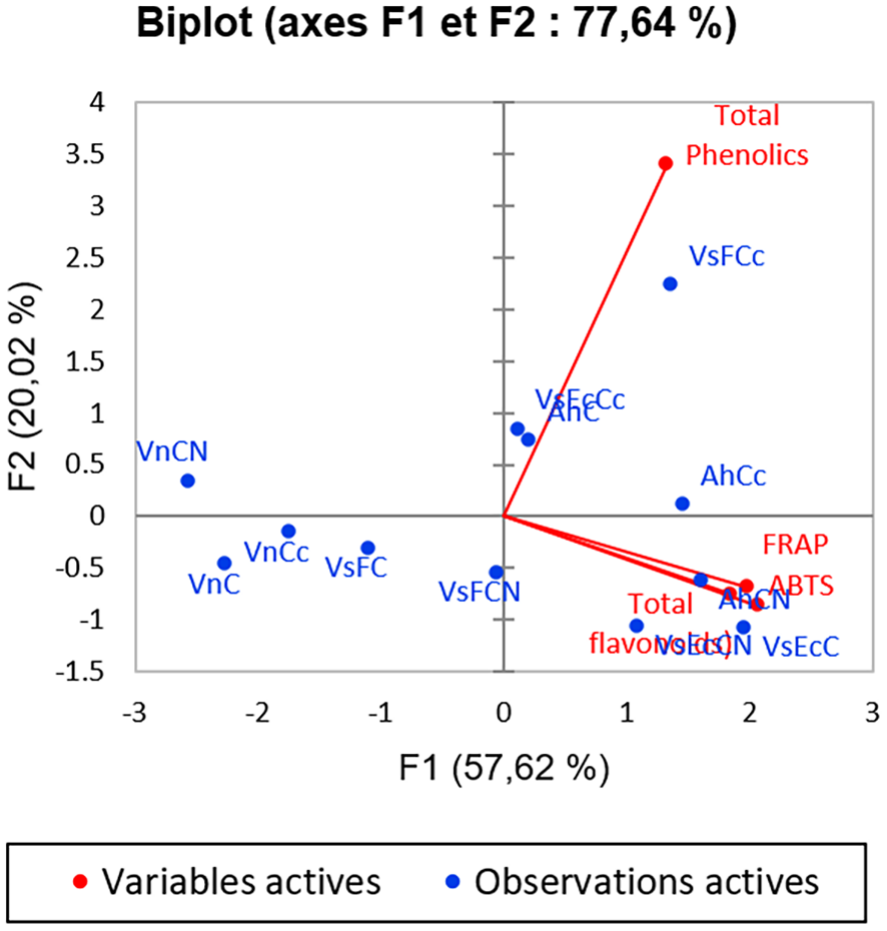
Principal component analysis (PCA) of the phytochemical profiles of aqueous decoctions and their antioxidant activities.

- Methanolic macerates (**Figure 5**): The first two principal components (PC1 and PC2) explained 39.82% and 29.07% of the total variance, respectively. PC1 was strongly associated with total polyphenols and FRAP reducing activity, whereas PC2 was moderately linked to flavonoids and strongly correlated with ABTS radical scavenging. This structuring highlights the contribution of phenolic compounds to antioxidant potential.
- Hydroalcoholic macerates (**Figure 6**): PC1 and PC2 accounted for 59.96% and 25.54% of the variance. PC1 was strongly influenced by polyphenols, flavonoids, and both ABTS and FRAP activities, while PC2 was primarily driven by ABTS activity, showing negligible correlation with other variables. This indicates that hydroalcoholic extracts capture broader chemodiversity but with ABTS as a discriminant factor.
- Aqueous decoctions (**Figure 7**): PC1 and PC2 explained 57.62% and 20.02% of the variance. PC1 was strongly linked to polyphenols, flavonoids, and FRAP activity, while PC2 was mainly influenced by flavonoid content alone. This suggests that decoctions reflect traditional preparations with distinct clustering based on flavonoid variability.

## 4 Discussion

The integrative analysis revealed that chemodiversity, driven by species identity and ecological origin, strongly modulates both phytochemical profiles and bioactivities. This highlights the ecological regulation of secondary metabolism as a key determinant of pharmacological potential.

The observed variability underscores the ecological and biochemical diversity of *Acanthospermum hispidum*, *Vitex simplicifolia*, and *Vetiveria nigritana*, three medicinal species widely used in Burkina Faso.

The quantitative assays demonstrated significant differences in total polyphenol and flavonoid contents across species and regions. *Vitex simplicifolia* consistently exhibited the highest concentrations of phenolic compounds, particularly in leaves collected from the Centre and Cascades regions, while *Vetiveria nigritana* rhizomes showed comparatively lower levels. This pattern suggests that environmental factors such as soil composition, rainfall, and temperature gradients modulate secondary metabolite biosynthesis (Alami et al., 2024; Qaderi et al., 2023; Yang et al., 2018). Specifically, humidity and temperature are known to stimulate phenylpropanoid and flavonoid biosynthesis, while nutrient availability influences terpenoid pathways, leading to distinct chemotypes across regions. The elevated polyphenol and flavonoid contents in *V. simplicifolia* from the southern regions (Centre and Cascades) may reflect higher humidity and temperature, conditions known to stimulate phenolic metabolism (Naikoo et al., 2019). Furthermore, the significant difference in total polyphenol and flavonoid contents between the hydroalcoholic extracts and the methanolic extracts and aqueous decoctions could be attributed to the difference in solvent polarity as well as the extraction method (Lezoul et al., 2020).

The antioxidant assays (ABTS and FRAP) confirmed that extracts enriched in polyphenols and flavonoids generally displayed stronger radical scavenging and reducing capacities (Ouahiba et al., 2020; Gangwar et al., 2014). However, correlation analyses revealed distinct patterns depending on the extraction solvent. In methanolic macerates, a negative correlation was observed between polyphenol content and ABTS activity (r = –0.373), suggesting that radical scavenging in this fraction is not primarily driven by polyphenols but may involve other metabolites such as terpenoids or synergistic compound interactions (Ksibi et al., 2022). In contrast, hydroalcoholic macerates showed a strong positive correlation between polyphenols and FRAP values (r = 0.708), supporting the role of phenolics in reducing capacity and electron-donating activity (Muflihah et al., 2021; Han et al., 2018). These solvent-dependent relationships highlight how extraction polarity influences the recovery of specific phenolic subclasses, thereby modulating antioxidant outcomes. Overall, the data emphasize the multifunctional role of phenolic metabolites in oxidative stress modulation and pathogen inhibition, while also underscoring that their contribution varies with extraction method and ecological origin (Rao and Zheng, 2025; Patil et al., 2024).

Methanolic macerates exhibited the most pronounced antiplasmodial activity, with *A. hispidum* from the Centre region showing the lowest IC_50_ (15.71 µg/mL), followed by *V. simplicifolia* leaves from the Cascades (25.04 µg/mL). This regional pattern suggests that plants from semi-humid zones may accumulate specific bioactive compounds such as flavones and terpenoids with antimalarial potential (Sarwar et al., 2024; Yan et al., 2026). The moderate activity observed in *V. simplicifolia* bark and leaves from the Centre-North region (IC_50_ = 42–47 µg/mL) further supports the hypothesis that geographical origin modulates pharmacological potency, possibly through differential expression of biosynthetic enzymes (Wu et al., 2024; Bailie et al., 2016).

Across all extraction methods, the rhizomes of *V. nigritana* demonstrated the strongest antibacterial activity, with MIC values ranging from 1.5 to 3 mg/mL and MBC/MIC ratios ≤ 2, confirming a bactericidal effect. The bark and leaves of *V. simplicifolia* and the leafy stems of *A. hispidum* exhibited moderate activity (MIC 1.5–6 mg/mL). These results align with previous reports attributing antibacterial properties to terpenoids and phenolic acids, which disrupt bacterial membranes and interfere with enzymatic systems (Rempe et al., 2017). The regional consistency of *V. nigritana’s* antibacterial potency suggests that its root metabolites are less sensitive to environmental variation, possibly due to their stable essential oil composition (Karimi et al., 2020; Massalha et al., 2017).

The Principal Component Analysis (PCA) provided a comprehensive visualization of the relationships among phytochemical variables, biological activities, and regional origin. The first principal component (PC1, 47.8%) was strongly correlated with polyphenols, flavonoids, and antioxidant indices (ABTS/FRAP), while the second component (PC2, 28.9%) was associated with IC_50_ and MIC values. The PCA biplot revealed three distinct clusters:

- *Vitex simplicifolia* (Centre, Cascades) grouped with high polyphenol/flavonoid content and strong antioxidant and antibacterial activities.
- *Acanthospermum hispidum* (Centre) aligned with low IC_50_ values, indicating potent antiplasmodial activity.
- *Vetiveria nigritana* (Centre-North, Cascades) clustered near the MIC vector, confirming its dominant antibacterial profile.

This multivariate pattern demonstrates that phytochemical richness and bioactivity are region-dependent (Kang et al., 2025), with southern regions (Centre and Cascades) favoring antioxidant and antiplasmodial potency, while northern zones (Centre-North) enhance antibacterial efficacy.

The combined evidence from correlation analysis and PCA highlights the existence of regional chemotypes with distinct therapeutic profiles (Lenka et al., 2025; Meng et al., 2023). Such differentiation has critical implications for the standardization and valorization of medicinal plants in Burkina Faso. Selecting optimal harvest regions could improve reproducibility and efficacy of phytomedicines (Zhang et al., 2025). Moreover, the strong correlations between phenolic content and biological activities support the use of polyphenol and flavonoid levels as biomarkers of pharmacological quality (E.A et al., 2025; Stachelska et al., 2025; Dominguez-López et al., 2024; Fathi Hafshejani et al., 2023).

Overall, the study demonstrates that the biological potential of *A. hispidum*, *V. simplicifolia*, and *V. nigritana* is intrinsically linked to their phytochemical composition and regional origin. The integration of quantitative assays, correlation analysis, and PCA provides a robust framework for understanding how ecological variability shapes medicinal efficacy (Srisittiratkul et al., 2025; Zhou et al., 2025). These findings not only validate traditional uses but also pave the way for the development of region-specific phytotherapeutic formulations, contributing to sustainable exploitation of Burkina Faso’s botanical resources and the global fight against oxidative stress, microbial infections, and malaria. Altogether, these findings demonstrate that ecological modulation of secondary metabolism generates region-specific chemotypes. This chemodiversity provides a scientific basis for rational selection of harvest regions to optimize phytomedicine standardization and biodiversity conservation.

This study has several methodological limitations that should be acknowledged. First, antioxidant activity was assessed using only ABTS and FRAP assays; inclusion of complementary methods such as DPPH would have provided a broader evaluation of radical scavenging mechanisms. Second, spectrophotometric approaches cannot separate or quantify individual compounds, which limits the ability to attribute bioactivity to specific metabolites. Third, plant materials were collected within a single year and from one site per region, which does not allow us to disentangle temporal variation from spatial variability. Fourth, the relatively small number of biological replicates (n = 3 per bioassay) reduces statistical power and may limit generalizability. These constraints mean that our findings should be interpreted as exploratory rather than definitive. Future work should integrate chromatographic and metabolomic techniques to resolve compound-specific contributions, expand sampling across multiple years and sites, and increase replication to strengthen statistical robustness. Such improvements will enhance reproducibility and provide a more comprehensive understanding of regional chemodiversity in medicinal plants.

## 5 Conclusion

The comprehensive evaluation of *Acanthospermum hispidum*, *Vitex simplicifolia*, and *Vetiveria nigritana* demonstrates that ecological origin is a decisive driver of chemodiversity, shaping both phytochemical richness and biological efficacy. By integrating quantitative assays, correlation analysis, and PCA, this study revealed coherent region-specific chemotypes in which polyphenol and flavonoid abundance consistently aligned with antioxidant, antiplasmodial, and antibacterial activities. Extracts from the Centre and Cascades regions were characterized by enhanced phenylpropanoid metabolism and superior antioxidant/antiplasmodial properties, whereas those from the Centre-North exhibited stronger antibacterial potency, reflecting distinct ecological adaptations. Strong positive correlations between phenolic content and antioxidant indices, together with inverse associations with IC_50_ and MIC values, confirm that phenolic metabolites are reliable biomarkers of pharmacological potential. The PCA further delineated ecological clustering of metabolite profiles, providing a mechanistic basis for targeted valorization and regional standardization of phytomedicines in Burkina Faso. Beyond validating traditional uses, these findings emphasize the need to integrate ecological parameters into phytochemical and pharmacological research to ensure reproducibility and optimize therapeutic outcomes. They also open promising avenues for the development of region-specific nutraceuticals and antimicrobial formulations, thereby contributing to sustainable exploitation of local biodiversity and advancing global efforts against oxidative stress, microbial infections, and malaria.

## Data Availability

The original contributions presented in the study are included in the article/supplementary material. Further inquiries can be directed to the corresponding author.
